# Registration Quality Assessment of Acupuncture Clinical Trials

**DOI:** 10.1371/journal.pone.0059506

**Published:** 2013-03-28

**Authors:** Jing Gu, Ye Zhao, Xiaogang Wang, Jianjun Jiang, Jinhui Tian, Kehu Yang

**Affiliations:** 1 Evidence-Based Medicine Center, Institute of Integrated Traditional Chinese and Western Medicine, School of Basic Medical Sciences, Lanzhou University, Lanzhou, Gansu, China; 2 Institute of Integrated Traditional Chinese and Western Medicine, Gansu University of Traditional Chinese Medicine, Lanzhou, Gansu, China; 3 Second School of Clinical Medicine of Lanzhou University, Lanzhou, Gansu, China; University of Chieti, Italy

## Abstract

**Background:**

Registration can help with transparency of acupuncture clinical trials (ACTs) by making protocol information and results available to the public. Recently, the number of registered ACTs has increased greatly, but only a few researchers have focused on the quality of ACTs registration. This review provides the first assessment of the registration quality of ACTs and the baseline information for future development.

**Methods:**

All records of ACTs registered in the World Health Organization (WHO) International Clinical Trials Registry Platform (ICTRP) were collected. Data was extracted and input to Excel spreadsheets. The current 20 items of the WHO Trial Registration Data Set (TRDS) and the special prepared items for acupuncture intervention details were used to assess the registration quality of ACTs.

**Results:**

A total of 740 records, found in 11 registries, were examined. The number of registered ACTs increased rapidly and involved a number of different diseases. The completeness of 20 items was not too poor due to 16 of them had a higher reported percentage (>85%). The completeness of the 20 items was different among registries. For example, the average registration percentage of 20 items in Clinicaltrials.gov, ChiCTR, ISRCTN and ANZCTR were 89.6%, 92.2%, 82.4% and 91.6% respectively. Detailed information regarding acupuncture intervention was seriously insufficient. Among the 740 registration records, 89.2% lacked information on the style of acupuncture, 80.8% did not contain details regarding the needles used, 53.5% lacked information on the treatment regimen and 76.2% did not give details of other interventions administered with acupuncture.

**Conclusions:**

The overall registration quality of ACTs is not high enough due to the serious lack of information on the specifics of acupuncture intervention. It is vital that a number of special items be set regarding acupuncture in order to develop a suitable system for the registration of ACTs.

## Introduction

As an alternative medicine methodology, acupuncture has proved effective for the treatment of many diseases and symptoms of disease, such as chronic pain, drug addiction, stroke rehabilitation, asthma and chemotherapy-induced nausea and vomiting [1], [2]. Acupuncture has been shown to provide definite curative effects and to cause fewer adverse reactions than some other treatment modalities and has been approved by the WHO and by many other medical and health institutions in some western countries [3]–[6], and is now practiced widely around the world.

Due to the availability of increased research funding, acupuncture clinical trials (ACTs) have been extensively conducted in many countries in recent decades. As a result, the number of papers related to acupuncture is growing rapidly. Many researchers have not only summarized the clinical efficacy and safety of acupuncture, but have also expressed concern regarding the quality of ACTs. These studies revealed that ACTs had many problems, such as poor methodological quality, unscientific research protocols, and repeated trials of acupuncture [7]. Moreover, the report quality of ACTs was low [8]. Information regarding acupuncturists’ qualifications, adverse reactions and follow-up was missing [9]. Problems with publication bias had also extended to ACTs. Vickers reported that all ACTs from mainland China, Hong Kong, Taiwan and Japan were reported to be effective [10]. Additionally, all acupuncture treatments reported in China and Russia (or the former Soviet Union) were reported to be valid. Obviously, the high proportion of positive results reported in these countries was unusual, which could be attributed to publication and selective reporting bias [11]. Based on the above findings, the results of acupuncture trails were found to be unconvincing and unreliable.

The protocol of study is a predefined written document to state the structure of a research project and guide the implementation. A typical protocol has the following elements: background information and scientific rationale, objectives, study design, study population, study procedure, statistical consideration, subject confidentiality, informed consent process, literature references and supplements/appendices. WHO International Clinical Trials Registry Platform (ICTPR) has put key protocol information about each trial in public domain. These information make us learn about general characteristics of trails before it is reported. The registration of clinical trial required that soon after the trial was completed, its salient data be entered onto registries. This would bring negative data to light. Recently, the number of registered ACTs has been greatly increasing. According to a single web portal provided by the WHO International Clinical Trials Registry Platform (ICTPR), the number of registered ACTs has climbed to 740 [12], 520 of which were registered after 2008.

Thus far researchers have focused mainly on the quality of trial registration [13]–[15], however, to our knowledge, no study has yet examined the registration quality of ACTs. It has also been unclear whether existing general registered items reflect the characteristics of acupuncture intervention and are suitable for ACTs. Therefore, the objective of this study is to summarize and evaluate the quality of ACTs registration with the current 20 items of the WHO trial registration data set (TRDS) [16] and the special prepared items for acupuncture intervention details, which will provide the baseline information for the future development of ACTs registration.

## Methods

### Source of Data

Using the ICTRP Search Portal (http://apps.who.int/trialsearch/Default.aspx), we researched the registration data sets of WHO registries. Registries included the following: Australian New Zealand Clinical Trials Registry (ANZCTR), Chinese Clinical Trial Register (ChiCTR), ClinicalTrials.gov, Clinical Trials Registry-India (CTRI), Cuban Public Registry of Clinical Trials (RPCEC), EU Clinical Trials Register (EU-CTR), German Clinical Trials Register (DRKS), Iranian Registry of Clinical Trials (IRCT), ISRCTN, Japan Primary Registries Network (JPRN), Pan African Clinical Trial Registry (PACTR), Sri Lanka Clinical Trials Registry (SLCTR), the Netherlands National Trial Register (NTR), Clinical Research Information Service (CRiS) public of Korea (KCT), Brazilian Clinical Trials Registry (ReBec).

### Search Strategy

WHO ICTRP prodives us with ‘standard search’ and ‘advanced search’. We selected ‘standard search’ (to input “acupuncture”, “needling”, “acupressure”, “moxibustion”, “auriculotherapy” and “acupoint” into the ICTRP Search Portal (http://apps.who.int/trialsearch/Default.aspx)) without any restriction to focus on the sensitivity of retrieval, expand the scope of search results and improve the recall ration. The search was conducted on data sets from the inception of the registries up to 23 July 2012.

### Inclusion Criteria

All records of ACTs that were registered in the WHO ICTRP were included. Acupuncture interventions might have been administered singly or with other interventions. For trials with multiple records, data from the record with the earliest registration date was chosen.

### Data Extraction and Analysis

Information of registered ACTs was collected from all of the chosen records which imported into the portal and additional information was viewed through a hyperlink to the record in the source registry (i.e. the registry that provided the data) that was published by ICTRP. A small pilot project designed to test the assessment of framework, and the training and competence of the data extractors, was performed prior to data extraction. Three researchers (Jing GU, Xiaogang WANG and Ye Zhao) extracted the data from 20 random records independently. Results of the pilot were discussed by Kehu Yang and Jing GU and the framework was subsequently adapted.

Two researchers (Jing GU and Ye ZHAO) extracted the data from each record independently. Disagreements were settled through discussion after data extraction. Data was input into a standardized form that was mainly composed of two parts: (1) the minimum 20 items of the WHO TRDS (Text S1) and (2) information related to acupuncture interventions: 1) styles of acupuncture, 2) needling details, 3) treatment regimen, and 4) other interventions administered along with the acupuncture.

Each item was assessed as “yes” (described in records) or ‘‘no’’ (not described in records). Conditions that were studied were classified according to the International Classification of Diseases (ICD-10) by two researchers (Jing GU and Ye Zhao). Discrepancies were resolved by consulting relevant clinical experts.

Descriptive statistics (frequency, percentage) were used to summarize data. Analysis was performed by Microsoft EXCEL software (version Microsoft Excel 2007; http://office.microsoft.com/zh-cn/) and SPSS ware (version 13.0; http://www.spss.com).

## Results

**Figure 1:**
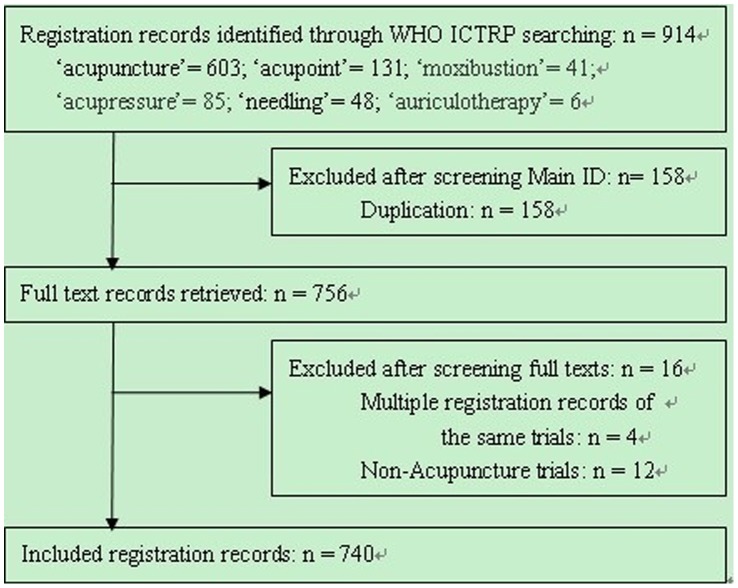
Flow chart of registration records identified, included and excluded.

A total of 914 registration records were retrieved in the registries. We excluded 174 records and included 740 records of ACTs (Figure 1, Text S2). Information from the registration of 740 trials was collected manually from the registered records.

### General Characteristics

#### 1. Year distribution of registration

**Figure 2:**
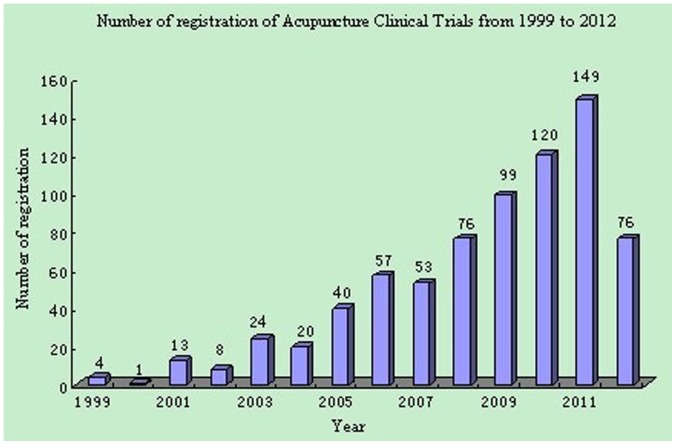
Number of registration of acupuncture clinical trials from 1999 to 2012.

All of the 740 acupuncture trials were registered during the period of 1999 to 2012. The number of registrations increased from 4 in 1999 to a peak of 149 in 2011. Registration for 2012 was still in process, so the final number was not yet available (Figure 2).

#### 2. The registration quantity of each registry

ACTs were found in the following 11 registries: ClinicalTrials.gov (376), ChiCTR (107), ISRCTN (100), ANZCTR (70), IRCT (46), JPRN (14), KCT (13), EU-CTR (4), DRKS (3), NTR (4) and ReBec (3); No registration records were found in CTRI, RPCEC, PACTR, and SLCTR.

#### 3. Types of diseases

Information was gathered concerning the diseases treated. Of these, 734 (99.2%, 95% CI 98.2–99.6%) records mentioned health condition(s) or problem(s), of which 38 (5.2%, 95% CI 3.8–7.0%) indicated that the participants were otherwise healthy people or did not specify. The most common conditions were “symptoms, signs and abnormal clinical and laboratory findings” (152 (20.7%, 95% CI 17.9–23.8%)). “Diseases of the musculoskeletal system and connective tissue” (100 (13.6%, 95% CI 11.3–16.3%) and “mental and behavioral disorders” (84 (11.4%, 95% CI 9.3–14.0%)) were of special concern (Table 1).

**Table 1:** Types of diseases.

Types of Diseases (Common ICD-10^a^)	Number (%) of n = 734, (95% CI)
Symptoms, signs and abnormal clinical and laboratory findings, not classified elsewhere	152 (20.7, 17.9–23.8)
Diseases of the musculoskeletal system and connective tissue	100 (13.6, 11.3–16.3)
Mental and behavioural disorders	84 (11.4, 9.3–14.0)
Diseases of the genitourinary system	50 (6.8, 5.2–8.9)
Diseases of the digestive system	46 (6.3, 4.7–8.3)
Diseases of the nervous system	45 (6.1, 4.6–8.1)
Pregnancy, childbirth and the puerperium	43 (5.9, 4.4–7.8)
Diseases of the circulatory system	41 (5.6, 4.1–7.5)
Diseases of the respiratory system	32 (4.4, 3.1–6.1)
Neoplasms	31 (4.2, 3.0–5.9)
Endocrine, nutritional and metabolic diseases	22 (3.0, 2.0–4.5)
Infectious and parasitic diseases	15 (2.0, 1.2–3.4)
Injury, poisoning and certain other consequences of external causes	14 (1.9, 1.1–3.2)
Diseases of the eye and adnexa	13 (1.8, 1.0–3.0)
Diseases of the skin and subcutaneous tissue	5 (0.7, 0.3–1.6)
Diseases of the ear and mastoid process	3 (0.4, 0.1–1.3)
Not specified or healthy people	38 (5.2, 3.8–7.0)

a Common ICD-10: International Classification of Diseases 10.

### 20 Items of WHO TRDS

The completeness of the 20 items of the WHO TRDS was checked. The overall report percentage of these 20 items varied from 38.6% to 100%. Information on the primary register and trial ID, date of registration in primary register and public title, was present in all registration records. Most registration records (>85%) mentioned the following items: source(s) of monetary or material support, primary sponsor, contact for public queries, scientific title, countries of recruitment, health condition(s) or problem(s) studied, intervention(s), key inclusion and exclusion criteria, study type, target sample size, recruitment status, primary outcome(s) and date of first enrollment. More than half of registration records provided items of secondary ID, key secondary outcomes and contact for scientific queries. Less than half of registration records provided information of secondary sponsor(s) (Table 2).

**Table 2:** 20 items of WHO TRDS.

20 Items	Overall	Clinical Trials.gov	ChiCTR	ISRCTN	ANZCTR	IRCT	JPRN	EU-CTR	DRKS	ReBec	KCT	NTR
	N^a^ (%) of n = 740, (95% CI)	N^a^ (%) of n = 376, (95% CI)	N^a^ (%) of n = 107, (95% CI)	N^a^ (%) of n = 100, (95% CI)	N^a^ (%) of n = 70, (95% CI)	N^a^ (%) of n = 46, (95% CI)	N^a^ (%) of n = 14, (95% CI)	N^a^ (%) of n = 4, (95% CI)	N^a^ (%) of n = 3, (95% CI)	N^a^ (%) of n = 3, (95% CI)	N^a^ (%) of n = 13, (95% CI)	N^a^ (%) of n = 4, (95% CI)
**Primary Register and Trial ID** b	740 (100.0, 98.9–100.0)	376 (100.0, 97.9–100.0)	107 (100.0, 93.0–100.0)	100 (100.0, 92.6–100.0)	70 (100.0, 89.7–100.0)	46 (100.0, 85.1–100.0)	14 (100.0, 63.4–100.0)	4 (100.0, 32.6–100.0)	3 (100.0, 26.6–100.0)	3 (100.0, 26.6–100.0)	13 (100.0, 61.6–100.0)	4 (100.0, 32.6–100.0)
**Date of Registration in Primary Registry**	740 (100.0, 98.9–100.0)	376 (100.0, 97.9–100.0)	107 (100.0, 93.0–100.0)	100 (100.0, 92.6–100.0)	70 (100.0, 89.7–100.0)	46 (100.0, 85.1–100.0)	14 (100.0, 63.4–100.0)	4 (100.0, 32.6–100.0)	3 (100.0, 26.6–100.0)	3 (100.0, 26.6–100.0)	13 (100.0, 61.6–100.0)	4 (100.0, 32.6–100.0)
**Secondary ID** b	445 (60.1, 56.6–63.6)	345 (91.8, 88.5–94.1)	0 (0.0, 0.0–7.0)	77 (77.0, 67.8–84.2)	10 (14.3, 7.9––24.6)	0 (0.0, 0–14.9)	0 (0.0, 0.0–36.6)	4 (100.0, 32.6–100.0)	3 (100.0, 26.6–100.0)	3 (100.0, 26.6–100.0)	0 (0.0, 0.0–38.4)	3 (75.0, 23.8–96.6)
**Source(s) of Monetary or Material Support**	730 (98.6, 97.5–99.3)	374 (99.5, 97.9–99.9)	106 (99.1, 93.7–99.9)	100 (100.0, 92.6–100.0)	68 (97.1, 89.3–99.3)	46 (100.0, 85.1–100.0)	14 (100.0, 63.4–100.0)	1 (25.0, 3.4–76.2)	3 (100.0, 26.6–100.0)	3 (100.0, 26.6–100.0)	12 (92.3, 60.9–98.9)	3 (75.0, 23.8–96.6)
**Primary Sponsor**	737 (99.6, 98.8–99.9)	374 (99.5, 97.9–99.9)	107 (100.0, 93.0–100.0)	100 (100.0, 92.6–100.0)	70 (100.0, 89.7–100.0)	46 (100.0, 85.1–100.0)	14 (100.0, 63.4–100.0)	4 (100.0, 32.6–100.0)	3 (100.0, 26.6–100.0)	3 (100.0, 26.6–100.0)	13 (100.0, 61.6–100.0)	3 (75.0, 23.8–96.6)
**Secondary Sponsor(s)**	286 (38.6, 35.2–42.2)	144 (38.3, 33.5–43.3)	95 (88.8, 81.3–93.5)	0 (0.0, 0.0–7.4)	27 (38.6, 28.0–50.4)	1 (2.2, 0.3–13.9)	0 (0.0, 0.0–36.6)	0 (0.0, 0.0–67.4)	1 (33.3, 4.3–84.6)	3 (100.0, 26.6–100.0)	13 (100.0, 61.6–100.0)	2 (50.0, 12.3–87.7)
**Contact for Public Queries**	711 (96.1, 94.4–97.3)	351 (93.4, 90.3–95.5)	107 (100.0, 93.0–100.0)	100 (100.0, 92.6–100.0)	70 (100.0, 89.7–100.0)	46 (100.0, 85.1–100.0)	14 (100.0, 63.4–100.0)	0 (0.0, 0.0–67.4)	3 (100.0, 26.6–100.0)	3 (100.0, 26.6–100.0)	13 (100.0, 61.6–100.0)	4 (100.0, 32.6–100.0)
**Contact for Scientific Queries**	397 (53.6, 50.0–57.2)	137 (36.4, 31.7–41.4)	107 (100.0, 93.0–100.0)	0 (0.0, 0.0–7.4)	70 (100.0, 89.7–100.0)	46 (100.0, 85.1–100.0)	14 (100.0, 63.4–100.0)	0 (0.0, 0.0–67.4)	3 (100.0, 26.6–100.0)	3 (100.0, 26.6–100.0)	13 (100.0, 61.6–100.0)	4 (100.0, 32.6–100.0)
**Public Title**	740 (100.0, 98.9–100.0)	376 (100.0, 97.9–100.0)	107 (100.0, 93.0–100.0)	100 (100.0, 92.6–100.0)	70 (100.0, 89.7–100.0)	46 (100.0, 85.1–100.0)	14 (100.0, 63.4–100.0)	4 (100.0, 32.6––100.0)	3 (100.0, 26.6–100.0)	3 (100.0, 26.6–100.0)	13 (100.0, 61.6––100.0)	4 (100.0, 32.6–100.0)
**Scientific Title**	638 (86.2, 83.5–88.5)	353 (93.9, 91.0–95.9)	106 (99.1, 93.7–99.9)	34 (34.0, 25.4–43.8)	70 (100.0, 89.7–100.0)	46 (100.0, 85.1––100.0)	2 (14.3, 3.6–42.7)	4 (100.0, 32.6–100.0)	3 (100.0, 26.6–100.0)	3 (100.0, 26.6–100.0)	13 (100.0, 61.6––100.0)	4 (100.0, 32.6–100.0)
**Countries of Recruitment**	723 (97.7, 96.3–98.6)	364 (96.8, 94.5–98.2)	103 (96.3, 90.5–98.6)	100 (100.0, 92.6––100.0)	70 (100.0, 89.7–100.0)	46 (100.0, 85.1–100.0)	14 (100.0, 63.4–100.0)	4 (100.0, 32.6–100.0)	2 (66.7, 15.4–95.7)	3 (100.0, 26.6–100.0)	13 (100.0, 61.6–100.0)	4 (100.0, 32.6–100.0)
**Health Condition(s) or Problem(s) Studied**	734 (99.2, 98.2–99.6)	374 (99.5, 97.9–99.9)	103 (96.3, 90.5–98.6)	99 (99.0, 93.2–99.9)	70 (100.0, 89.7–100.0)	46 (100.0, 85.1–100.0)	14 (100.0, 63.4–100.0)	4 (100.0, 32.6–100.0)	3 (100.0, 26.6–100.0)	3 (100.0, 26.6–100.0)	13 (100.0, 61.6–100.0)	4 (100.0, 32.6–100.0)
**Intervention(s)**	737 (99.6, 98.8–99.9)	376 (100.0, 97.9–100.0)	104 (97.2, 91.7–99.1)	100 (100.0, 92.6–100.0)	70 (100.0, 89.7–100.0)	46 (100.0, 85.1–100.0)	14 (100.0, 63.4–100.0)	4 (100.0, 32.6–100.0)	3 (100.0, 26.6–100.0)	3 (100.0, 26.6–100.0)	13 (100.0, 61.6–100.0)	4 (100.0, 32.6–100.0)
**Key Inclusion and Exclusion Criteria**	720 (97.3, 95.8–98.2)	374 (99.5, 97.9–99.9)	107 (100.0, 93.0–100.0)	82 (82.0, 73.2–88.4)	70 (100.0, 89.7–100.0)	46 (100.0, 85.1–100.0)	14 (100.0, 63.4–100.0)	4 (100.0, 32.6–100.0)	3 (100.0, 26.6–100.0)	3 (100.0, 26.6–100.0)	13 (100.0, 61.6–100.0)	4 (100.0, 32.6–100.0)
**Study Type**	736 (99.5, 98.6–99.8)	376 (100.0, 97.9–100.0)	107 (100.0, 93.0–100.0)	99 (99.0, 93.2–99.9)	70 (100.0, 89.7–100.0)	46 (100.0, 85.1–100.0)	14 (100.0, 63.4–100.0)	4 (100.0, 32.6–100.0)	3 (100.0, 26.6–100.0)	0 (0.0, 0.0–73.4)	13 (100.0, 61.6–100.0)	4 (100.0, 32.6–100.0)
**Date of First Enrollment**	739 (99.9, 99.0–100.0)	376 (100.0, 97.9–100.0)	107 (100.0, 93.0–100.0)	100 (100.0, 92.6–100.0)	70 (100.0, 89.7–100.0)	46 (100.0, 85.1–100.0)	14 (100.0, 63.4–100.0)	4 (100.0, 32.6–100.0)	2 (66.7, 15.4–95.7)	3 (100.0, 26.6––100.0)	13 (100.0, 61.6–100.0)	4 (100.0, 32.6–100.0)
**Target Sample Size**	703 (95.0, 93.2–96.4)	352 (93.6, 90.7–95.7)	103 (96.3, 90.5–98.6)	95 (95.0, 88.5–97.9)	70 (100.0, 89.7–100.0)	46 (100.0, 85.1–100.0)	13 (92.9, 63.0–99.9)	1 (25.0, 3.4–76.2)	3 (100.0, 26.6–100.0)	3 (100.0, 26.6–100.0)	13 (100.0, 61.6–100.0)	4 (100.0, 32.6–100.0)
**Recruitment Status**	738 (99.7, 98.9–99.9)	376 (100.0, 97.9–100.0)	107 (100.0, 93.0–100.0)	100 (100.0, 92.6–100.0)	70 (100.0, 89.7–100.0)	46 (100.0, 85.1–100.0)	14 (100.0, 63.4–100.0)	4 (100.0, 32.6–100.0)	3 (100.0, 26.6–100.0)	3 (100.0, 26.6–100.0)	13 (100.0, 61.6–100.0)	4 (100.0, 32.6–100.0)
**Primary Outcome(s)**	677 (91.5, 89.2–93.3)	325 (86.4, 82.6–89.5)	106 (99.1, 93.7–99.9)	91 (91.0, 83.6–95.3)	70 (100.0, 89.7–100.0)	46 (100.0, 85.1––100.0)	13 (92.9, 63.0–99.9)	4 (100.0, 32.6–100.0)	3 (100.0, 26.6–100.0)	3 (100.0, 26.6–100.0)	13 (100.0, 61.6–100.0)	3 (75.0, 23.8–96.6)
**Key Secondary Outcomes**	495 (66.9, 63.4–70.2)	237 (63.0, 58.0–67.8)	78 (72.9, 63.7–80.5)	72 (72.0, 62.4–79.9)	58 (82.9, 72.2–90.0)	25 (54.3, 40.0–68.0)	4 (28.6, 11.1–56.1)	1 (25.0, 3.4–76.2)	1 (33.3, 4.3–84.6)	3 (100.0, 26.6–100.0)	13 (100.0, 61.6–100.0)	3 (75.0, 23.8–96.6)
**Total per Registry (%)**	(89.0, 86.5–91.0)	(89.6, 86.1–92.3)	(92.2, 85.4–96.0)	(82.4, 73.7–88.7)	(91.6, 82.5–96.2)	(87.8, 74.9–94.6)	(81.4, 53.3–94.4)	(73.8, 23.2–96.3)	(90.0, 17.2–99.7)	(95.0, 9.6–100.0)	(94.6, 61.3–99.5)	(91.2, 24.5–99.7)

a N = Number.

b ID = Identifying Number.

The four centers that had the largest number of registered ACTs were Clinical Trials.gov, ChiCTR, ISRCTN and ANZCTR, and the average registration rate of 20 items were 89.6%, 92.2%, 82.4%, and 91.6% respectively (Table 2).

Information regarding eligibility criteria for participants was also gathered. A total of 615 (83.1%, 95% CI 80.2–85.6%) records mentioned diagnostic criteria, of which 567 were based solely on “western medicine (diseases)”, 17 were based solely on “traditional medicine syndrome” and 31 used both “western disease” and “traditional medicine syndrome” diagnostic criteria. Exclusion criteria were reported in 693 (93.6%, 95% CI 91.6–95.2%) records, and most (>90%) provided information regarding the age and sex of participants (Table 3).

**Table 3:** Eligibility criteria for participants.

Category	Number (%) of n = 740, (95% CI)
**Diagnostic Criteria**	615 (83.1, 80.2–85.6)
Western Medicine (diseases)	567 (76.6, 73.4–79.5)
Traditional Medicine	17 (2.3, 1.4–3.7)
Using both Disease and Syndrome	31 (4.2, 3.0–5.9)
**Exclusion Criteria**	693 (93.6, 91.6–95.2)
Age	682 (92.2, 90.0–93.9)
Sex	679 (91.8, 89.5–93.5)
Male	14 (2.1^a^, 1.2–3.5)
Female	145 (21.4^a^, 18.4–24.6)
Both	520 (76.6^a^, 73.2–79.6)

a n = 679.

### Intervention Specifics of ACTs

Quality of registration of acupuncture intervention specifics was also checked. These intervention specifics are shown in Tables 4 and 5.

**Table 4:** Special items for acupuncture intervention details.

Special Items for Acupuncture Intervention Details	Overall	Clinical Trials.gov	ChiCTR	ISRCTN	ANZCTR	IRCT	JPRN	EU-CTR	DRKS	ReBec	KCT	NTR
	N^a^ (%) of n = 740, (95% CI)	N^a^ (%) of n = 376, (95% CI)	N^a^ (%) of n = 107, (95% CI)	N^a^ (%) of n = 100, (95% CI)	N^a^ (%) of n = 70, (95% CI)	N^a^ (%) of n = 46, (95% CI)	N^a^ (%) of n = 14, (95% CI)	N^a^ (%) of n = 4, (95% CI)	N^a^ (%) of n = 3, (95% CI)	N^a^ (%) of n = 3, (95% CI)	N^a^ (%) of n = 13, (95% CI)	N^a^ (%) of n = 4, (95% CI)
**Style of Acupuncture** b	80 (10.8, 8.8–13.3)	42 (11.2, 8.4–14.8)	12 (11.2, 6.5–18.7)	8 (8.0, 4.1–15.2)	11 (15.7, 8.9–26.2)	4 (8.7, 3.3–21.0)	0 (0.0, 0.0–36.6)	0 (0.0, 0.0–67.4)	0 (0.0, 0.0–73.4)	0 (0.0, 0.0–73.4)	5 (38.5, 17.0–65.6)	0 (0.0, 0.0–67.4)
**Needling Details** c	(19.2, 16.5–22.2)											
**Names or Location of Points**	216 (29.2, 26.0–32.6)	95 (25.3, 21.1–29.9)	28 (26.2, 18.7–35.3)	16 (16.0, 10.0–24.5)	36 (51.4, 39.9–62.9)	29 (63.0, 48.4–75.6)	5 (35.7, 15.7–62.4)	0 (0.0, 0.0–67.4)	0 (0.0, 0.0–73.4)	1 (33.3, 4.3–84.6)	9 (69.2, 40.9–88.8)	0 (0.0, 0.0–67.4)
**Number of Needle Insertions per Session**	169 (22.8, 20.0–26.0)	82 (21.8, 17.9–26.3)	17 (15.9, 10.1–24.1)	15 (15.0, 9.2–23.4)	27 (38.6, 28.0–50.4)	19 (41.3, 28.1–55.9)	3 (21.4, 7.1–49.4)	0 (0.0, 0.0–67.4)	0 (0.0, 0.0–73.4)	1 (33.3, 4.3–84.6)	5 (38.5, 17.0–65.6)	0 (0.0, 0.0–67.4)
**Depth of Insertion**	47 (6.4, 4.8–8.4)	25 (6.6, 4.5–9.7)	3 (2.8, 0.9–8.3)	7 (7.0, 3.4–14.0)	5 (7.1, 3.0–16.0)	2 (4.3, 1.1–15.8)	1 (7.1, 1.0–37.0)	0 (0.0, 0.0–67.4)	0 (0.0, 0.0–73.4)	1 (33.3, 4.3–84.6)	3 (23.1, 7.6–52.2)	0 (0.0, 0.0–67.4)
**Response Sought**	58 (7.8, 6.1–10.0)	27 (7.2, 5.0–10.3)	6 (5.6, 2.5–11.9)	4 (4.0, 1.5–10.2)	15 (21.4, 13.4–32.6)	2 (4.3, 1.1–15.8)	0 (0.0, 0.0–36.6)	0 (0.0, 0.0–67.4)	0 (0.0, 0.0–73.4)	1 (33.3, 4.3–84.6)	3 (23.1, 7.6–52.2)	0 (0.0, 0.0–67.4)
**Needle Retention Time**	216 (29.2, 26.0–32.6)	95 (25.3, 21.1–29.9)	21 (19.6, 13.2–28.2)	15 (15.0, 9.2–23.4)	43 (61.4, 49.6–72.0)	28 (60.9, 46.2–73.8)	2 (14.3, 3.6–42.7)	0 (0.0, 0.0–67.4)	0 (0.0, 0.0–73.4)	1 (33.3, 4.3–84.6)	11 (84.6, 54.9–96.1)	0 (0.0, 0.0–67.4)
**Needle Type** d	84 (11.4, 9.3–13.8)	50 (13.3, 10.2–17.1)	3 (2.8, 0.9–8.3)	7 (7.0, 3.4–14.0)	15 (21.4, 13.4–32.6)	1 (2.2, 0.3–13.9)	0 (0.0, 0.0–36.6)	0 (0.0, 0.0–67.4)	0 (0.0, 0.0–73.4)	1 (33.3, 4.3–84.6)	7 (53.8, 28.2–77.6)	0 (0.0, 0.0–67.4)
**Needle Stimulation**	205 (27.7, 24.6–31.0)	101 (26.9, 22.6–31.6)	30 (28.0, 20.4–37.3)	17 (17.0, 10.8–25.7)	33 (47.1, 35.8–58.8)	10 (21.7, 12.1–35.9)	5 (35.7, 15.7–62.4)	1 (25.0, 3.4–76.2)	0 (0.0, 0.0–73.4)	2 (66.7, 15.4–95.7)	4 (30.8, 12.0–59.1)	0 (0.0, 0.0–67.4)
**Treatment Regimen** e	344 (46.5, 42.9–50.1)	184 (48.9, 43.9–54.0)	18 (16.8, 10.9–25.1)	38 (38.0, 29.0–47.9)	54 (77.1, 65.9–85.5)	30 (65.2, 50.5–77.5)	3 (21.4, 7.1–49.4)	0 (0.0, 0.0–67.4)	1 (33.3, 4.3–84.6)	3 (100.0, 26.6–100)	12 (92.3, 60.9–98.9)	1 (25.0, 3.4–76.2)
**Other Interventions** f	176 (23.8, 20.9–27.0)	80 (21.3, 17.4–25.7)	28 (26.2, 18.7–35.3)	25 (25.0, 17.5–34.4)	21 (30.0, 20.4–41.7)	8 (17.4, 8.9–31.1)	1 (7.1, 1.0–37.0)	0 (0.0, 0.0–67.4)	1 (33.3, 4.3–84.6)	0 (0.0, 0.0–73.4)	4 (30.8, 12.0–59.1)	0 (0.0, 0.0–67.4)

a N = Number.

b Style of acupuncture (e.g. Traditional Chinese Medicine,Japanese,Korean,Western medical,Five Element,ear acupuncture, etc).

c Needling details can contain seven items: names or location of points used (uni/bilateral), number of needle insertions per subject per session, depth of insertion, response sought (e.g. de qi or muscle twitch response), needle retention time, needle type (diameter, length, and manufacturer or material) and needle stimulation (e.g. manual, electrical).The total percentage of the seven items was 19.2%.

d Needle Type (e.g. diameter, length, and manufacturer or material).

e Treatment regimen, refers to number, frequency and duration of treatment sessions.

f Other interventions administered to the acupuncture group (e.g. moxibustion, cupping, herbs, exercises, lifestyle advice).

**Table 5:** Descriptive information of some acupuncture special items.

Category	Number (%) of n, (95% CI)
Style of Acupuncture	n = 97
Traditional Chinese Medicine Acupuncture	33 (34.0, 25.3–44.0)
Ear Acupuncture	28 (28.9, 20.7–38.6)
Moxibustion	14 (14.4, 8.7–22.9)
Western Style Acupuncture	13 (13.4, 7.9–21.7)
Japanese Medicine Acupuncture	5 (5.2, 2.2–11.8)
Korean Medical Acupuncture	2 (2.1, 0.5–7.9)
Tongue Acupuncture	2 (2.1, 0.5–7.9)
**Needle Stimulation of the Acupuncture**	**n = 309**
Electrical	108 (35.0, 29.8–40.4)
Manual	46 (14.9, 11.3–19.3)
Laser	26 (8.4, 5.8–12.1)
Not specified or other stimulation	129 (41.7, 36.4–47.3)
**Details of other Interventions Administered to Acupuncture**	**n = 176**
Western Medicine	67 (38.1, 31.2–45.5)
Traditional Chinese Medicine	13 (7.4, 4.3–12.3)
Exercises, Rehabilitation, Massage,Moxibustion, Cupping, etc.	37 (21.0, 15.6–27.7)
Cognitive Behavioural Therapy, Life Style Advice, etc.	9 (5.1, 2.7–9.5)
Other Interventions (Routine nursing, Conventional Therapy, etc.)	50 (28.4, 22.2–35.5)

Style of acupuncture was reported in 80 (10.8%, 95% CI 8.8–13.3%) trials (Table 4). They covered the following seven types: “traditional Chinese medicine acupuncture”, “ear acupuncture”, “moxibustion”, “western style acupuncture” “Japanese medicine acupuncture”, “Korean medical acupuncture” and “tongue acupuncture” (Table 5). The lowest reported percentage of this item was 0% from JPRN, EU-CTR, DRKS, ReBec and NTR while the highest was 38.5% from KCT (Table 4).

Details regarding needling could potentially contain seven items: names or location of points used (uni/bilateral), number of needle insertions per subject per session, depth of insertion, response sought (e.g. de qi or muscle twitch response), needle retention time, needle type (diameter, length, and manufacturer or material) and needle stimulation (e.g. manual, electrical). The average report percentage of these seven items was 19.2% and the lowest reported percentage was 0% (Table 4). Regarding needle stimulation, 35.0% had electrical stimulation, 14.9% had manual stimulation and 8.4% had laser stimulation (Table 5).

Treatment regimen, which refers to information of number, frequency and duration of treatment sessions, was present in 344 (46.5%, 95% CI 42.9–50.1%) trials (Table 4). EU-CTR had the lowest reported percentage (0%) of this item while ReBec, KCT and ANZCTR had the higher reported percentage (>75%) (Table 4).

Details of other interventions administered along with acupuncture were reported in 176 (23.8%, 95% CI 20.9–27.0%) trials (Table 4). They covered the following five types interventions: “western medicine”, “traditional Chinese medicine”, “exercises rehabilitation, massage, moxibustion, cupping, or similar”, “cognitive behavioral therapy, life style advice, or similar”, and “other interventions (routine nursing, conventional therapy, etc.)” (Table 5). EU-CTR, ReBec and NTR had the lowest reported percentage (0%) of this item (Table 4).

## Discussion

A small number of studies have assessed the registration quality of clinical trials, and only one study was related to ACTs [17]. However, that study only investigated the status quo of the registration for ACTs, but did not report on its quality. In the present study, we retrieved all registered records of ACTs and evaluated the overall quality in order to identify possible deficiencies in ACTs registration and to provide feasible recommendations for development.

We reviewed some of the same variables reported in previous research done by Yang et. al. (2008) [17]. In that study, 206 registration records of ACTs were searched between 1999 and 2008 [17]. In the current study, results retrieved from the WHO ICTRP indicated that the registration number of ACTs has been increasing rapidly in recent years and has reached 740 as of July, 2012. In our research, there are 11 register centers where we can find ACTs while there were only 3 in the Yang study [17]. Similar to previous studies, ClinicalTrials.gov still have the largest number of ACTs while the number of ACTs in ChiCTR has increased from just 5, four years ago [17], to 107, surpassing ISRCTN and ANZCTRN and making it the second biggest registration center. This rapid increase in the number of registration centers that have ACTs indicates that ACTs registration has become much more common. Although the number of ACTs in ChiCTR is far less than in ClinicalTrials.gov, the rapid growth is obvious, considering it has only been four years since it was established. Therefore it is possible that ChiCTR will become the principle registration center for ACTs in the future. Previous studies reported that the diseases of registered ACTs were limited [17], [18]. In contrast, our study found that the types of diseases studied with ACTs are becoming more varied which will be helpful to better develop acupuncture clinical research.

Each registration record was also analyzed and the quality of ACT registration assessed using the following two guidelines: (1) the completeness of 20 items of WHO TRDS and (2) whether the related information of acupuncture intervention was present in the registration records according to specially preparedspecific items.

The contents of clinical trial registrations should contain the basic information about the trial’s implementation. The 20 items of the WHO TRDS [16], regarded as the minimum requirement for trial registration, comprehensively covers this basic information. Therefore, ensuring the completeness of the 20 items is the first step in assessing the quality of ACT registration. Complete registration of 20 items minimizes the chance that relevant information will be omitted. This is of importance since missing or uninformative items required by the WHO TRDS may impair the fulfillment of registration promise. In the current study 20 items of the 740 records were reviewed and appraised for completeness. Our results indicated that most items (16 items) had higher percentages (>85%) of completeness. However, information regarding secondary sponsor(s), contact for scientific queries, secondary ID and key secondary outcomes were insufficient. There are two possible reasons for the omission of relevant information. One is that not every participant is willing to provide all 20 items due to academic or commercial interests [19]; the other is that not every registered trial had all of the 20 items [14]. For example, secondary ID or secondary sponsors do not routinely exist in some clinical trials so that the total percentage of these corresponding entries might be lower.

The completeness of 20 items varied among the 11 registries. Clinicaltrials.gov, the center with the largest number of registered ACTs, did not have the highest registration quality while instead we found that ChiCTR had the highest in the registration quality. The completeness of the 20 items in different register centers may be related to the establishment time of centers. Specifically, the 20 items of the WHO TRDS was announced in 2007 so that those centers established after this announcement would have set the items according to the WHO TRDS. Thus, the items from those centers would have a higher percentage of registration than the centers established prior to 2007. For example, ChiCTR, which was established after 2007, was found to have better completeness and quality of the 20 items.

In the current study we also have determined that for each register center, there were several items that contained woefully inadequate information. This may have been caused by the absence of related items in that specific center. For instance, ISRCIN has no area for information about ‘secondary sponsor(s)’ [14], and no record contained relevant information. Similarly, the item ‘secondary sponsor’ was not found in IRCT [14], and only 2.2% records provided relevant information. This speculation is supported by previous research by Liu’s [14]. Liu mentioned that, although the data sets of each registry were based on the 20 items, there were still slight differences among registries due to the increase or reduction of some items so that there are different registration rates in different centers.

Unlike general clinical intervention, acupuncture is a special medical technique that treats patients by inserting thin needles into acupoints. Acupuncture clinical practice is mainly concerned with acupuncture theory, acupoints, unique acupuncture manipulation methods, and acupuncture instruments [20]. The “quality” of an intervention in the sense of “how well made is the intervention” is a preclinical not a clinical issue for drugs, biologics, or devices, but for procedures such as acupuncture, intervention quality is a clinical issue. There are many factors which can influence its curative effect and safety. Arguably more information thoroughly describing the acupuncture intervention should be required in the registration of ACTs, just as in the registration of drug, biological and vaccine trials, information on the dose, frequency, route of administration and duration of treatment are needed [16], [21]. As an open-source, the information of ACTs registration should be detailed enough to make trials as transparent as possible.

Since acupuncture is a unique medical technique, it is not appropriate to evaluate the quality of its intervention specifics with existing evaluation criterion for general intervention measures. As demonstrated by previous studies, special items that describe the intervention details of acupuncture should be added to the registration of item sets [17]. Further assessment on the quality of ACTs registration could potentially be accomplished using these special items. We developed items and evaluation criterion that contain the following four facets: style of acupuncture, needling details, treatment regimen and other interventions administered with acupuncture. We validated these items for quality evaluation of acupuncture intervention details in reference to the Standards for Reporting Interventions in Controlled Trials of Acupuncture (STRICTA) [22]. As is known to all, the intervention details of acupuncture are totally included by STRICTA. However, unlike the trial report, trial registration is a demonstration of the protocol before implementation while the trial report is the demonstration of the process and details about the trial after completion. Therefore, instead of using all of the items published by STRICTA, we integrated the suggestions of intervention from acupuncturists, clinical expert, methodologists and producers of systematic reviews and chose only those items related with intervention that should be included in a protocol of acupuncture research (the protocol without these information will miss the accuracy of the implementation of trails and the results will be unreliable.). However, we found that information on the details of acupuncture intervention is seriously insufficient. Possible reasons for this may be: (1) these special items were not included in 20 items and the registration centers lacked registration requirements for them. Therefore, these items failed to catch the attention of registrants and registrants neglected the description of acupuncture intervention details or (2) the selection of acupoints and acupuncture manipulation takes great skill and heavy reliance on experience, therefore many clinical acupuncture practitioners keep this information private and are reluctant to share technical details. These limitations cause ACTs to be less transparent.

Acupuncture is a part of Traditional Chinese Medicine (TCM). The necessity of acupuncture intervention and selection of acupoints are based on TCM’s syndromes. However, most acupuncture researchers focus on ‘western disease’ for their diagnostic criteria rather than ‘TCMs’ syndromes’. In the current study, we found that only 6.5% (2.3% + 4.2%) of ACTs registration records provided information about TCM’s syndrome in their diagnostic criteria. To comply with the principles of selection of treatment based on the differential diagnosis of TCM, we strongly propose that diagnostic criteria contain information on both western disease and TCM’s syndrome in the registration of ACTs.

After the publication of the announcement of International Committee of Medical Journal Editors (ICMJE) [23], the Ottawa Statement and Declaration of Helsinki [24], [25], and with the establishment of the WHO ICTRP, the importance of registration has been gradually accepted so that now, clinical trial registration has become a part of the current research paradigm internationally. With increasing numbers and attention in recent years, ACTs should be encouraged to register in order to improve public confidence in clinical effectiveness. Registration could bring the following benefits to the clinical research of acupuncture. First, registration promotes the inspection of study design and provides technical guidance for the clinical trial [26]. Second, whether the key part of clinical trial was reported or not, a registered protocol can be used as a basis for supervision. Registration will also increase the transparency of the clinical trials, so it can be ensured that the reported information from clinical research is complete, true and valid [24], [27] while potential biases are reduced as much as possible. Third, registration of ACTs helps to avoid repeating research and/or wasting resources. Fourth, as a precondition of ACTs being published in journals with high impact factors [18], registration could facilitate the communication of the results from ACTs. Therefore, further quantitative and qualitative improvements are needed in ACTs registration.

Although the quality of ACTs registration was fair in regard to the completeness of the 20 items according to our research, the serious lack of information on acupuncture intervention specifics lowered its overall quality greatly, which made trial registration fail to fulfill its promise of promoting research transparency [28]–[30].

It is very necessary to establish special registration system for ACTs because of the specificity of acupuncture, just like STRICTA – a special version of CONSORT [31] – is made for acupuncture RCTs. Modifying the existing registration system to include criteria (or items) specific to acupuncture could facilitate developing a registration system for ACTs. The first step would be to establish a registration data set suitable for ACTs. We propose that those items used to assess acupuncture intervention details in the current study be added on the basis of the 20 items. The second step would be to establish a special registry center for ACTs.

There were several limitations in the current study. First, assessment of the registration quality of ACTs in different centers according to the completeness of 20 items may be lacking fairness since the 20 items of the WHO TRDS were not announced until 2007. Therefore, those centers established after 2007 may have a higher registration quality since they could more easily meet the standards set by the WHO TRDS. Second, there are likely many clinical trials that are not registered in the WHO ICTRP, so that it would be impossible for our study to include all of the ACTs that are registered throughout the world.

In conclusion, the overall registration quality of ACTs was not high enough due to the serious lack of information on the specifics of acupuncture intervention. Further improvements are needed in this field. This could be achieved by optimizing the WHO TRDS to include those additional items related to acupuncture. It is extremely vital to establish a number of special items regarding acupuncture intervention to develop a suitable system for the registration of ACTs.

## Supporting Information

Text S1 **The minimum 20 items of the WHO trial registration data set (WHO TRDs).**
(DOC)

Text S2 **Main ID of all included registration records.**
(DOC)
