# Supplementary material for: Registration Quality Assessment of Acupuncture Clinical Trials
Source: PLoS One. 2013 Mar 28;8(3):e59506. doi: 10.1371/journal.pone.0059506 (PMC3610907; doi:10.1371/journal.pone.0059506)
Supplement: Text S1 — The minimum 20 items of the WHO trial registration data set (WHO TRDs). (DOC) [file pone.0059506.s001.doc]

Text S1 The minimum 20 items of the WHO trial registration data set (WHO TRDs).

(1) Primary Registry and Trial Identifying Number;

(2) Date of Registration in Primary Registry;

(3) Primary Sponsor(s);

(4) Public Title;

(5) Source(s) of Monetary or Material Support;

(6) Scientiﬁc Title;

(7) Date of First Enrollment;

(8) Target Sample Size;

(9) Recruitment Status;

(10) Study Type;

(11) Countries of Recruitment;

(12) Key Inclusion and Exclusion Criteria;

(13) Secondary Sponsor(s);

(14) Health Condition(s) or Problem(s) Studied;

(15) Intervention(s);

(16) Primary Outcome(s);

(17) Key Secondary Outcomes;

(18) Secondary Identifying Numbers;

(19) Contact for Public Queries;

(20) Contact for Scientiﬁc Queries.
