# Supplementary material for: Registration Quality Assessment of Acupuncture Clinical Trials
Source: PLoS One. 2013 Mar 28;8(3):e59506. doi: 10.1371/journal.pone.0059506 (PMC3610907; doi:10.1371/journal.pone.0059506)
Supplement: Text S2 — Main ID of all included registration records. (DOC) [file pone.0059506.s002.doc]

Text S2 Main ID of all included registration records.

**(bold：the increased registration records)**

| ClinicalTrials.gov (376 records) | | | | | | | |
| --- | --- | --- | --- | --- | --- | --- | --- |
| NCT01651338 | NCT01274793 | NCT00935701 | NCT00599677 | NCT00279071 | NCT00002149 | | |
| NCT01650363 | NCT01271595 | NCT00935155 | NCT00599586 | NCT00275574 | NCT00000817 | | |
| NCT01650207 | NCT01270607 | NCT00932139 | NCT00598988 | NCT00261755 | NCT00000394 | | |
| NCT01643577 | NCT01266044 | NCT00932061 | NCT00591877 | NCT00260637 | **NCT01617837** | | |
| NCT01634022 | NCT01259180 | NCT00921492 | NCT00571480 | NCT00260494 | **NCT01614444** | | |
| NCT01633996 | NCT01250093 | NCT00917215 | NCT00570024 | NCT00253994 | **NCT01608633** | | |
| NCT01633281 | NCT01247935 | NCT00913354 | NCT00554879 | NCT00245752 | **NCT01608048** | | |
| NCT01631500 | NCT01231776 | NCT00907621 | NCT00549835 | NCT00244894 | **NCT01582724** | | |
| NCT01627444 | NCT01231425 | NCT00900965 | NCT00547690 | NCT00233857 | **NCT01545960** | | |
| NCT01621139 | NCT01227096 | NCT00892268 | NCT00545623 | NCT00225316 | **NCT00017823** | | |
| NCT01619176 | NCT01218243 | NCT00891618 | NCT00544401 | NCT00221247 | **NCT01516502** | | |
| NCT01614639 | NCT01205958 | NCT00885586 | NCT00528554 | NCT00219505 | **NCT00220688** | | |
| NCT01604356 | NCT01201642 | NCT00885183 | NCT00528125 | NCT00217074 | **NCT01513824** | | |
| NCT01598974 | NCT01197950 | NCT00883831 | NCT00523016 | NCT00209001 | **NCT01509326** | | |
| NCT01612663 | NCT01189994 | NCT00882271 | NCT00521547 | NCT00200889 | **NCT01424072** | | |
| NCT01580280 | NCT01178008 | NCT00874133 | NCT00508482 | NCT00200733 | **NCT01420848** | | |
| NCT01579786 | NCT01174394 | NCT00868517 | NCT00502619 | NCT00200096 | **NCT01420835** | | |
| NCT01573858 | NCT01165099 | NCT00862550 | NCT00493753 | NCT00186654 | **NCT00069732** | | |
| NCT01572168 | NCT01163682 | NCT00860301 | NCT00487331 | NCT00177840 | **NCT01389570** | | |
| NCT01570127 | NCT01163409 | NCT00859365 | NCT00487266 | NCT00177450 | **NCT01378793** | | |
| NCT01551654 | NCT01162317 | NCT00855140 | NCT00470795 | NCT00148577 | **NCT01366456** | | |
| NCT01526031 | NCT01162018 | NCT00843765 | NCT00464425 | NCT00142597 | **NCT01354860** | | |
| NCT01512433 | NCT01152632 | NCT00839592 | NCT00460161 | NCT00142532 | **NCT01346267** | | |
| NCT01495286 | NCT01149317 | NCT00838994 | NCT00459680 | NCT00136669 | **NCT01339728** | | |
| NCT01492738 | NCT01143688 | NCT00828516 | NCT00455182 | NCT00104546 | **NCT01317082** | | |
| NCT01492569 | NCT01141231 | NCT00826397 | NCT00449241 | NCT00103675 | **NCT01301170** | | |
| NCT01491321 | NCT01124955 | NCT00826345 | NCT00432289 | NCT00094874 | **NCT01290653** | | |
| NCT01487590 | NCT01116453 | NCT00826215 | NCT00430378 | NCT00093327 | **NCT01281904** | | |
| NCT01486303 | NCT01112943 | NCT00815529 | NCT00425776 | NCT00090389 | **NCT00003817** | | |
| NCT01483885 | NCT01105221 | NCT00815464 | NCT00425412 | NCT00090337 | **NCT01189110** | | |
| NCT01481090 | NCT01102868 | NCT00813683 | NCT00421902 | NCT00086021 | **NCT01167348** | | |
| NCT01479920 | NCT01102816 | NCT00804089 | NCT00419822 | NCT00081965 | **NCT01156129** | | |
| NCT01457209 | NCT01096420 | NCT00803582 | NCT00417313 | NCT00079898 | **NCT01141374** | | |
| NCT01449396 | NCT01094782 | NCT00797732 | NCT00414505 | NCT00071669 | **NCT01074164** | | |
| NCT01447329 | NCT01088867 | NCT00786214 | NCT00405158 | NCT00070967 | **NCT01056458** | | |
| NCT01442012 | NCT01079689 | NCT00780884 | NCT00404443 | NCT00070824 | **NCT01013558** | | |
| NCT01439412 | NCT01079390 | NCT00779818 | NCT00402961 | NCT00067691 | **NCT00995917** | | |
| NCT01422928 | NCT01060553 | NCT00768014 | NCT00401063 | NCT00065975 | **NCT00978185** | | |
| NCT01417741 | NCT01055561 | NCT00758017 | NCT00388752 | NCT00065585 | **NCT00974870** | | |
| NCT01411501 | NCT01054495 | NCT00746200 | NCT00379496 | NCT00065403 | **NCT00959998** | | |
| NCT01408212 | NCT01052857 | NCT00736411 | NCT00379327 | NCT00065234 | **NCT00892216** | | |
| NCT01398930 | NCT01050075 | NCT00732459 | NCT00375102 | NCT00064818 | **NCT00890474** | | |
| NCT01395511 | NCT01049074 | NCT00724763 | NCT00369902 | NCT00064740 | **NCT00046865** | | |
| NCT01389622 | NCT01047384 | NCT00722462 | NCT00360841 | NCT00060021 | **NCT00832468** | | |
| NCT01389349 | NCT01043692 | NCT00714727 | NCT00355329 | NCT00059345 | **NCT00788970** | | |
| NCT01376375 | NCT01040754 | NCT00694447 | NCT00353847 | NCT00055354 | **NCT00670917** | | |
| NCT01369238 | NCT01037894 | NCT00692328 | NCT00352248 | NCT00040833 | **NCT00629655** | | |
| NCT01368393 | NCT01030029 | NCT00689897 | NCT00346736 | NCT00035399 | **NCT00550251** | | |
| NCT01360229 | NCT01022177 | NCT00685789 | NCT00335946 | NCT00034047 | **NCT00526565** | | |
| NCT01357343 | NCT01018108 | NCT00682162 | NCT00334165 | NCT00034034 | **NCT00457717** | | |
| NCT01350570 | NCT01017211 | NCT00674713 | NCT00333606 | NCT00032422 | **NCT00430313** | | |
| NCT01345994 | NCT01014221 | NCT00658034 | NCT00328796 | NCT00029497 | **NCT00310063** | | |
| NCT01345409 | NCT01013337 | NCT00656513 | NCT00325663 | NCT00011024 | **NCT00277407** | | |
| NCT01339715 | NCT01005108 | NCT00655317 | NCT00322504 | NCT00010985 | **NCT00275483** | | |
| NCT01338701 | NCT01003951 | NCT00646633 | NCT00320138 | NCT00010972 | **NCT00247156** | | |
| NCT01331395 | NCT01003470 | NCT00641303 | NCT00317317 | NCT00010946 | **NCT00243269** | | |
| NCT01315561 | NCT01002846 | NCT00639977 | NCT00317291 | NCT00010764 |  | | |
| NCT01304979 | NCT00969280 | NCT00636012 | NCT00312585 | NCT00010647 |  | | |
| NCT01301482 | NCT00969267 | NCT00635037 | NCT00307918 | NCT00010621 |  | | |
| NCT01285687 | NCT00965367 | NCT00624793 | NCT00307788 | NCT00010517 |  | | |
| NCT01283477 | NCT00957112 | NCT00621660 | NCT00302185 | NCT00010504 |  | | |
| NCT01276028 | NCT00952432 | NCT00610584 | NCT00297427 | NCT00010491 |  | | |
| NCT01275989 | NCT00950482 | NCT00608660 | NCT00286390 | NCT00010478 |  | | |
| NCT01275807 | NCT00945074 | NCT00602940 | NCT00284492 | NCT00005770 |  | | |
| ChiCTR (107 records) | | | | | | | |
| ChiCTR-TRC-12002206 | ChiCTR-TRC-11001347 | ChiCTR-TRC-10000887 | ChiCTR-TRC-08000184 | ChiCTR-OCH-11001546 | | **ChiCTR-TRC-10001005** | |
| ChiCTR-TRC-12002081 | ChiCTR-TRC-11001343 | ChiCTR-TRC-10000807 | ChiCTR-TRC-08000176 | ChiCTR-OCH-09000335 | | **ChiCTR-TRC-10000993** | |
| ChiCTR-TRC-12002003 | ChiCTR-TRC-11001310 | ChiCTR-TRC-10000792 | ChiCTR-TRC-08000143 | **ChiCTR-TRC-12002338** | | **ChiCTR-TRC-09000709** | |
| ChiCTR-TRC-12001972 | ChiCTR-TRC-11001257 | ChiCTR-TRC-10000791 | ChiCTR-TRC-08000138 | **ChiCTR-TRC-12002272** | | **ChiCTR-TRC-09000692** | |
| ChiCTR-TRC-12001971 | ChiCTR-TRC-11001256 | ChiCTR-TRC-10000746 | ChiCTR-TRC-08000088 | **ChiCTR-TRC-12002059** | | **ChiCTR-TRC-09000604** | |
| ChiCTR-TRC-11001813 | ChiCTR-TRC-11001245 | ChiCTR-TRC-09000527 | ChiCTR-TRC-08000083 | **ChiCTR-TRC-11001790** | | **ChiCTR-TRC-09000602** | |
| ChiCTR-TRC-11001727 | ChiCTR-TRC-11001201 | ChiCTR-TRC-09000519 | ChiCTR-TRC-08000069 | **ChiCTR-TRC-11001780** | | **ChiCTR-TRC-09000601** | |
| ChiCTR-TRC-11001719 | ChiCTR-TRC-11001169 | ChiCTR-TRC-09000507 | ChiCTR-TRC-07000036 | **ChiCTR-TRC-11001775** | | **ChiCTR-TRC-09000600** | |
| ChiCTR-TRC-11001693 | ChiCTR-TRC-10001609 | ChiCTR-TRC-09000471 | ChiCTR-TRC-07000024 | **ChiCTR-TRC-11001586** | | **ChiCTR-TRC-09000599** | |
| ChiCTR-TRC-11001655 | ChiCTR-TRC-10001160 | ChiCTR-TRC-09000455 | ChiCTR-TRC-07000010 | **ChiCTR-TRC-11001409** | | **ChiCTR-TRC-09000528** | |
| ChiCTR-TRC-11001600 | ChiCTR-TRC-10001146 | ChiCTR-TRC-08000302 | ChiCTR-TNRC-10001073 | **ChiCTR-TRC-11001408** | | **ChiCTR-TRC-09000484** | |
| ChiCTR-TRC-11001554 | ChiCTR-TRC-10001138 | ChiCTR-TRC-08000297 | ChiCTR-TNRC-07000035 | **ChiCTR-TRC-11001401** | | **ChiCTR-TRC-09000464** | |
| ChiCTR-TRC-11001540 | ChiCTR-TRC-10001086 | ChiCTR-TRC-08000283 | ChiCTR-TCH-11001547 | **ChiCTR-TRC-11001382** | | **ChiCTR-ONRC-08000255** | |
| ChiCTR-TRC-11001388 | ChiCTR-TRC-10001078 | ChiCTR-TRC-08000278 | ChiCTR-ORC-09000505 | **ChiCTR-TRC-11001306** | | **ChiCTR-TNRC-11001299** | |
| ChiCTR-TRC-11001379 | ChiCTR-TRC-10001023 | ChiCTR-TRC-08000276 | ChiCTR-ORC-08000305 | **ChiCTR-TRC-11001300** | | **ChiCTR-TNRC-11001292** | |
| ChiCTR-TRC-11001353 | ChiCTR-TRC-10000950 | ChiCTR-TRC-08000261 | ChiCTR-ONRC-11001311 | **ChiCTR-TRC-11001248** | | **ChiCTR-TNRC-10000883** | |
| ChiCTR-TRC-11001352 | ChiCTR-TRC-10000902 | ChiCTR-TRC-08000225 | ChiCTR-ONRC-08000277 | **ChiCTR-TRC-10001025** | | **ChiCTR-ORC-08000304** | |
| ChiCTR-TRC-11001349 | ChiCTR-TRC-10000889 | ChiCTR-TRC-08000209 | ChiCTR-ONRC-08000260 | **ChiCTR-TRC-10001007** | |  | |
| ISRCTN (100 records) | | | | | | | |
| ISRCTN99754128 | ISRCTN84496835 | ISRCTN61111416 | ISRCTN39740785 | ISRCTN18747033 | ISRCTN05305406 | | |
| ISRCTN99496264 | ISRCTN83626912 | ISRCTN60217348 | ISRCTN36780548 | ISRCTN18249834 | ISRCTN05293321 | | |
| ISRCTN99395260 | ISRCTN81864187 | ISRCTN59953486 | ISRCTN34841555 | ISRCTN18207278 | ISRCTN02971192 | | |
| ISRCTN98703707 | ISRCTN81672591 | ISRCTN59267538 | ISRCTN34405634 | ISRCTN16856737 | ISRCTN01382777 | | |
| ISRCTN98448646 | ISRCTN80764175 | ISRCTN59155637 | ISRCTN32823720 | ISRCTN15186354 | **ISRCTN08714168** | | |
| ISRCTN97373659 | ISRCTN80312467 | ISRCTN52683557 | ISRCTN30663359 | ISRCTN14801647 | **ISRCTN88229446** | | |
| ISRCTN97151578 | ISRCTN78434638 | ISRCTN52062666 | ISRCTN29932220 | ISRCTN13737091 | **ISRCTN87604299** | | |
| ISRCTN96537534 | ISRCTN76896018 | ISRCTN50179298 | ISRCTN29230777 | ISRCTN13130687 | **ISRCTN82622830** | | |
| ISRCTN84841116 | ISRCTN74414962 | ISRCTN49884134 | ISRCTN28687220 | ISRCTN12585433 | **ISRCTN77598683** | | |
| ISRCTN94142364 | ISRCTN74335441 | ISRCTN49839714 | ISRCTN27450856 | ISRCTN12159894 | **ISRCTN75722066** | | |
| ISRCTN93327878 | ISRCTN74318483 | ISRCTN49335612 | ISRCTN26438275 | ISRCTN11374571 | **ISRCTN01897462** | | |
| ISRCTN92205535 | ISRCTN74259594 | ISRCTN48408850 | ISRCTN25134802 | ISRCTN09754699 | **ISRCTN30907460** | | |
| ISRCTN90807007 | ISRCTN71727409 | ISRCTN43104115 | ISRCTN24863192 | ISRCTN08827905 | **ISRCTN22726482** | | |
| ISRCTN88597683 | ISRCTN66117475 | ISRCTN41920953 | ISRCTN23891318 | ISRCTN08236707 | **ISRCTN12864149** | | |
| ISRCTN84709751 | ISRCTN65814467 | ISRCTN41571810 | ISRCTN23245449 | ISRCTN07857866 | **ISRCTN10634508** | | |
| ISRCTN88008690 | ISRCTN63787732 | ISRCTN40932605 | ISRCTN22866867 | ISRCTN07165558 |  | | |
| ISRCTN84985339 | ISRCTN61381178 | ISRCTN40706107 | ISRCTN22366060 | ISRCTN06223266 |  | | |
| ANZCTR (70 records) | | | | | | | |
| ACTRN12612000719831 | ACTRN12608000264381 | ACTRN12609000985280 | ACTRN12610000611022 | ACTRN12611000288921 | | | **ACTRN12607000585426** |
| ACTRN12605000314628 | ACTRN12609000073202 | ACTRN12609000989246 | ACTRN12610000626066 | ACTRN12611000393954 | | | **ACTRN12608000149369** |
| ACTRN12605000367640 | ACTRN12609000095268 | ACTRN12609001001280 | ACTRN12610000631000 | ACTRN12611000614998 | | | **ACTRN12609000470291** |
| ACTRN12605000483651 | ACTRN12609000101280 | ACTRN12609001054202 | ACTRN12610000720011 | ACTRN12611000761965 | | | **ACTRN12610000824066** |
| ACTRN12605000766617 | ACTRN12609000288224 | ACTRN12610000043033 | ACTRN12610000850077 | ACTRN12612000032853 | | | **ACTRN12611000668909** |
| ACTRN12606000494538 | ACTRN12609000326291 | ACTRN12610000104055 | ACTRN12610001052022 | ACTRN12612000096853 | | | **ACTRN12611000899943** |
| ACTRN12607000015448 | ACTRN12609000480280 | ACTRN12610000232033 | ACTRN12611000025932 | ACTRN12612000607875 | | | **ACTRN12611001126909** |
| ACTRN12607000105448 | ACTRN12609000676213 | ACTRN12610000335099 | ACTRN12611000088943 | ACTRN12612000667819 | | | **ACTRN12611001204932** |
| ACTRN12607000499482 | ACTRN12609000698279 | ACTRN12610000340033 | ACTRN12611000096954 | ACTRN12612000693820 | | | **ACTRN12612000719831** |
| ACTRN12608000050358 | ACTRN12609000751279 | ACTRN12610000344099 | ACTRN12611000113954 | ACTRN12612000705886 | | | **ACTRN12612000758808** |
| ACTRN12608000231347 | ACTRN12609000923268 | ACTRN12610000368033 | ACTRN12611000149965 | ACTRN12612000706875 | | |  |
| ACTRN12608000239369 | ACTRN12609000928213 | ACTRN12610000370000 | ACTRN12611000226909 | **ACTRN12606000419561** | | |  |
| IRCT (46 records) | | | | | | | |
| IRCT201202088956N1 | IRCT201106036699N1 | IRCT138811163284N1 | **IRCT201112118374N1** | **IRCT201104216247N1** | **IRCT138901253706N1** | | |
| IRCT201201127117N2 | IRCT201105243217N3 | IRCT138706101061N2 | **IRCT201111146575N4** | **IRCT201103192699N4** | **IRCT138812193527N1** | | |
| IRCT201201108235N1 | IRCT201104104422N2 | **IRCT201206079965N1** | **IRCT201109257631N1** | **IRCT201102014853N1** | **IRCT138811203311N1** | | |
| IRCT201112014578N4 | IRCT201101133004N4 | **IRCT201204065698N3** | **IRCT138804232170N1** | **IRCT201102011310N6** | **IRCT138810162437N3** | | |
| IRCT201111218151N1 | IRCT201011275181N4 | **IRCT201201038100N2** | **IRCT201108276247N2** | **IRCT138904174347N1** | **IRCT138808272731N1** | | |
| IRCT201108174242N2 | IRCT138905104450N1 | **IRCT201112278537N1** | **IRCT201108037217N1** | **IRCT138904144321N1** | **IRCT138807152556N1** | | |
| IRCT201107267117N1 | IRCT138902222891N1 | **IRCT201112268273N2** | **IRCT201106145864N2** | **IRCT138904104281N1** |  | | |
| IRCT201107026934N1 | IRCT138902013767N1 | **IRCT201112214613N8** | **IRCT201106104641N4** | **IRCT138904071788N3** |  | | |
| JPRN (14 records) | | | | | | | |
| JPRN-UMIN000007773 | JPRN-UMIN000006167 | JPRN-UMIN000005644 | JPRN-UMIN000002588 | **JPRN-UMIN000006269** | | |  |
| JPRN-UMIN000006957 | JPRN-UMIN000005645 | JPRN-UMIN000003115 | JPRN-UMIN000002254 | **JPRN-UMIN000005913** | | |  |
| JPRN-UMIN000001277 | JPRN-UMIN000000994 | JPRN-UMIN000002237 | JPRN-UMIN000002140 |  | | |  |
| KCT (13 records) | | | | | | | |
| KCT0000385 | KCT0000195 | KCT0000164 | KCT0000071 | **KCT0000469** |  | | |
| KCT0000383 | KCT0000169 | KCT0000153 | KCT0000019 | **KCT0000402** |  | | |
| KCT0000269 | KCT0000168 | KCT0000130 |  |  |  | | |
| NTR (4 records) | | | | | | | |
| NTR3132 | NTR1987 | NTR1613 | **NTR2554** |  |  | | |
| ReBec (3 records) | | | | | | | |
| RBR-5g7xqh | RBR-59f4yr | RBR-58yq52 |  |  |  | | |
| EU-CTR (4 records) | | | | | | | |
| EUCTR2008-002343-16-IT | EUCTR2006-004698-86-DE | EUCTR2006-000947-24-GB | EUCTR2006-000810-18-GB |  |  | | |
| DRKS (3 records) | | | | | | | |
| DRKS00003767 | DRKS00003116 | DRKS00000164 |  |  |  | | |
